# Natural selection and human adiposity: crafty genotype, thrifty phenotype

**DOI:** 10.1098/rstb.2022.0224

**Published:** 2023-09-11

**Authors:** Jonathan C. K. Wells

**Affiliations:** Childhood Nutrition Research Centre, Population Policy and Practice Department, UCL Great Ormond Street Institute of Child Health, 30 Guilford Street, London WC1N 1EH, UK

**Keywords:** adiposity, evolution, infectious disease, fat distribution, phenotypic plasticity, reaction norm

## Abstract

Evolutionary perspectives on obesity have aimed to understand how the genetic constitution of individuals has been shaped by selective pressures such as famine, predation or infectious disease. The dual intervention model assumes strong selection on lower and upper limits of adiposity, but negligible fitness implications for intermediate adiposity. These frameworks are agnostic to age, sex and condition. I argue that selection has favoured a ‘crafty genotype’—a genetic basis for accommodating variability in the ‘fitness value’ of fat through phenotypic plasticity, depending on the endogenous and exogenous characteristics of each individual. Hominin evolution occurred in volatile environments. I argue that the polygenetic basis of adiposity stabilizes phenotype in such environments, while also coordinating phenotypic variance across traits. This stability underpins reaction norms through which adiposity can respond sensitively to ecological factors. I consider how the fitness value of fat changes with age, sex and developmental experience. Fat is also differentially distributed between peripheral and abdominal depots, reflecting variable prioritization of survival versus reproduction. Where longevity has been compromised by undernutrition, abdominal fat may promote immediate survival and fitness, while long-term cardiometabolic risks may never materialize. This approach helps understand the sensitivity of adiposity to diverse environmental factors, and why the health impacts of obesity are variable.

This article is part of a discussion meeting issue ‘Causes of obesity: theories, conjectures and evidence (Part I)’.

## 1. Introduction

Although obesity became a global health epidemic only recently, it has attracted interest from evolutionary biologists for over half a century [1–3]. Body mass index (BMI), the most widely used index of nutritional status, shows substantial variability both within and across populations [4]. Although an inaccurate marker of adiposity at the individual level, BMI trends reflect enormous population variability in body fatness, ranging in adults from below 5% of body weight to over 50% [5]. At a proximate level, the explanation for such variability lies in the interaction between our genes, physiology and environment [6]. At an ultimate level, we need an evolutionary framework to understand *why* humans are differentially susceptible to obesity [5].

From an evolutionary perspective, our susceptibility to excess weight must broadly derive from the human genome. While fatness is sensitive to environmental factors, obesity has substantial heritability, demonstrated by twin and adoption studies [7]. To date, around 60 genome-wide association studies (GWAS) have linked over 1100 loci with adiposity phenotypes [8], though they explain only a small minority of the heritability. Like height, adiposity appears to be a quintessential polygenetic trait, whereby numerous alleles each contribute a very small magnitude of effect to phenotypic variance [9]. Increasing GWAS sample size will likely allow many more alleles to be identified. Most evolutionary perspectives on obesity have therefore focused on explaining why individuals might differ in alleles associated with BMI and adiposity.

In 1962, Neel published his ‘thrifty genotype’ hypothesis of diabetes susceptibility [1]. This approach assumed that ancestral humans experienced selective pressures from ‘cycles of feast and famine’, through periods of energy-plenty and energy-scarcity. Those more regularly exposed to such conditions were hypothesized to have evolved an enhanced capacity for fat accumulation during times of surplus, which could then be oxidized during famines. In contemporary settings of unlimited food availability, accordingly, thrifty genotypes would drive development of obesity and diabetes. Scientists have searched extensively for thrifty genes, but despite the evidence from GWAS [8,10], the hypothesis remains essentially unsupported [5,11].

An alternative perspective proposes that contemporary genetic variability in BMI emerged in the *absence* of strong selection. Speakman's ‘drifty genotype’ hypothesis assumes that the emergence of the capacity to use fire and weapons for defence among the genus *Homo* would have relaxed the selective pressure of predation, weakening upper constraints on body mass and allowing random mutations to accumulate [11].

A challenge for both thrifty and drifty models as originally proposed is that human adiposity is inherently variable within individuals in response to environmental factors; hence genotype has a variable association with phenotype [12]. Attention has therefore shifted to the regulation of body weight and adiposity.

In the short term, both diets and overfeeding interventions have only transient effects, with weight returning to its baseline level [11]. Whether explicitly or implicitly expressed, a common assumption is that weight is regulated around a set point, and that obesity develops when this regulatory system in some way fails. However, adiposity clearly varies over longer periods. One theoretical solution to this dilemma is Speakman's ‘dual intervention point’ model, which assumes that selection has shaped regulatory mechanisms to maintain fat stores above a lower level, necessary to buffer famine, and below an upper level that raises predation risk [11].

While the dual intervention point model clearly represents a conceptual advance, there are several outstanding issues. First, existing evolutionary models are agnostic over age, and yet human adiposity varies profoundly with age in a sex-specific manner, suggesting that fat stores have highly variable ‘fitness value’ through the life-course. Moreover, the sensitivity of adiposity to prevailing ecological conditions and to developmental trajectory indicates that each individual may alter its ‘fitness valuation’ of fat according to its experience. This requires that we pay more attention to the multiple reaction norms, through which a given genotype can give rise to multiple possible phenotypes through the life-course. These reaction norms relate both to developmental plasticity, through which early exposures may leave long-lasting effects on phenotype [13,14], and phenotypic flexibility through adult life, whereby fat stores may increase and decrease in response to successive exposures [15,16].

A second limitation is that most evolutionary models of obesity treat adipose tissue as a ‘petrol tank’, an inert fuel dump where energy is stored at time A for potential use at time B. Such a framework ignores how the metabolic activity of adipose tissue varies by anatomical depot, and offers minimal insight into fat distribution and how it varies with both endogenous and external factors.

Third, characterizing adiposity as a polygenetic trait, where many alleles each have negligible impact on phenotype, begs the question of *why* its genetic basis should have this profile. Such an ‘infinitesimal’ model helps explain why BMI demonstrates a normal distribution in populations such as hunter–gatherers (though in obesogenic settings, the distribution becomes right-skewed). What are widely termed ‘adiposity' or ‘obesity’ alleles are typically also associated with other physiological traits, and only appear relevant to obesity because they leave a signal in population BMI variability, more so among individuals with high compared with low BMI [17]. Despite their small effects, often equivalent to a few grams of weight [18], selection has clearly acted on adiposity alleles sufficiently strongly to generate the patterns of age and sex variability reviewed below. Paradoxically, minimal attention has been paid to the genetic basis of the reaction norms, through which adiposity can change over time within individuals, both during development and through adult life.

Understanding of this genetic and phenotypic variability in adiposity is currently missing from evolutionary debates on obesity. Optimal strategies for storing fat in unpredictable or changing environments have been explored using mathematical models [19,20], but these address only environmental variability, and do not consider endogenous factors such as age, sex or phenotypic condition. Below, I build on previous frameworks to present a ‘crafty genotype’ hypothesis, aiming to improve understanding both of the polygenic basis of adiposity and its variability with age, sex and life-course experience. First, I revisit the context in which human adiposity evolved.

## 2. Evolution of human adiposity

Compared with other extant apes, humans have both greater levels of body fatness, especially in females, and a unique life-course profile of adiposity [5,21]. This indicates that adiposity underwent strong selection in the evolution of our lineage; moreover, this evolutionary process connects with the emergence of other ‘quintessential human characteristics', such as our bipedal posture, large brains, sociality, reproductive demography and extended lifespans [5]. All of these traits demonstrate mosaic evolution from the australopithecines onwards [22], and adiposity is likely to have evolved in similar mosaic manner, meaning that different fat depots may have responded to different selective pressures in different periods of time.

The evolution of the genus *Homo* occurred in the context of the emergence of the savannah niche in Sub-Saharan Africa. While the global climate became overall cooler and more arid in this period, isotopic indices of palaeoclimate also indicate dramatically increasing climatic volatility [23]. Ecological change became so pronounced that it was often experienced across generations and within life-courses, such as transient but extreme El Niño Southern Oscillation (ENSO) events [24]. In other words, the hominin ecological niche was not occasionally disrupted, rather continual disruption *was* the niche [25–27].

In unpredictable environments, adaptive strategies cannot be elicited directly from the genome, but must instead emerge through plastic responses. Hominin evolution can therefore be considered a sorting process, selecting in favour of phenotypes that could survive and reproduce in increasingly unpredictable environments. Alongside other traits with low plasticity (skeletal anatomy, large brains), selection favoured both larger energy stores and also the capacity to acquire and use them in concert with diverse stimuli and threats [28–32].

**Figure 1.**
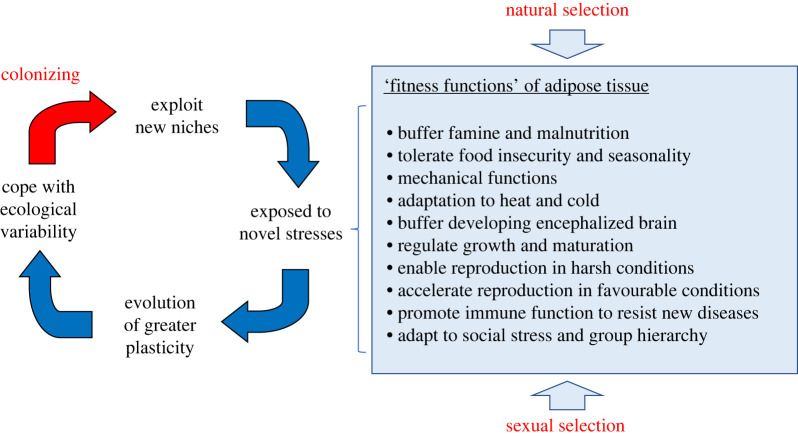
Schematic diagram illustrating the fitness functions of adipose tissue, and their exposure through human behaviour to the selective pressure of colonizing novel environments. (Online version in colour.)

Adiposity has a wide range of fitness functions that span the life-history functions of survival and maintenance, growth, reproduction and defence (figure 1). All of these functions can be assumed to have undergone stronger selection in volatile ecological niches. Importantly, humans themselves must have amplified these selective pressures, through their own colonizing behaviour which regularly exposed them to new ecological stresses requiring rapid accommodation [33–35]. The fact that contemporary humans demonstrate remarkably low levels of genetic diversity [36], despite having colonized a huge variety of ecological niches [37], indicates that plastic responses have been the key mode of geographic adaptation. As with other species exposed to volatile environments, human genetic evolution appears to have shifted from local differentiation to the enhancement of plasticity [24,38].

In this context of persistent ancestral exposure to ecological volatility [23], the evolution of the brain and adiposity in *Homo* are intricately connected. They act as complementary ‘risk management’ systems, whereby the brain processes acute signals that act on the nervous system, while adipose tissue processes environmental signals that act on energetics and physiological function [28]. Most mammals have specialized in only one of these systems [39], but humans have both, a phenotype consistent with the notion that hominins evolved under persistently high levels of ecological risk. Indeed, evolutionary change in adiposity and metabolism is likely to have preceded the encephalized *Homo* brain. The brain has obligate fuel requirements, preferentially metabolizing glucose or, during starvation, ketones obtained from fat oxidation [40]. Metabolic adaptations to ecological volatility may have favoured sufficient stabilizing of fuel supply at the level of tissues for substantial expansion of the brain to be viable [24]. As human encephalization is a developmental process, this may further have favoured greater fat stores at birth and in early infancy [41].

Producing encephalized neonates and infants is costly for mothers, especially in colonizing species where conditions at the time of conception may be unrelated to conditions at the time of late gestation and lactation, when reproductive costs are greatest. This selective pressure has favoured high levels of adiposity in females of reproductive age, as well as compensatory reductions in fat-free mass, resulting in major sexual dimorphism in adult human body composition [42]. Humans are quintessential ‘capital breeders’, storing fat in advance so that lactation can be funded regardless of immediate food supply. Colonizing new niches produces boom–bust population dynamics [33], for which fat is highly adaptive: females can use fat stores to ride out lean periods, and then produce offspring rapidly if conditions improve [33]. During the 1974–1975 Bangladesh famine, for example, fertility declined by 34%, but then increased above the long-term background rate by 17% after the famine [43].

However, hominin evolutionary change involved changes not only in adult size and body composition, but also in the tempo and trajectory of maturation, and in longevity [44,45]. Moreover, the *Homo* genus evolved not only novel developmental trajectories and life stages, but also substantial plasticity therein, so that the age at which developmental milestones are reached is impacted both by genetic constitution and developmental experience [33]. In contemporary humans, fat stores contribute to the regulation of maturation in both sexes, while the timing of menarche is also associated with adult adiposity [14,46]. More fundamentally, these evolutionary trends have left a signal in the association of adiposity with age.

Volatile niches impact every constituent species, and this includes pathogens. Globally, parasites and pathogens are most prevalent in the tropics where *Homo* evolved [47]; however, the distribution of many diseases also responds sensitively to short-term variation in climate, such as temperature and rainfall. Since infection was the primary cause of human mortality until the recent epidemic of non-communicable disease, immune response was under powerful selective pressure [48,49]. Fat stores play a double role in immune response, not only by supplying energy for costly metabolic processes, but also by secreting pro- and anti-inflammatory cytokines [50,51]. For example, the risk of death in malnourished children is greatest if they have low leptin, a hormone secreted by fat that regulates immune function [31]. Beyond ecological volatility, human sociality may itself have created new niches for infections, initially through larger social groups in ancestral hunter–gatherer populations, and subsequently through the emergence of agriculture. Sedentary farming populations amplified the selective pressure of infection, owing to dense settlements, population growth, pathogens jumping to humans from newly domesticated animals, and unhygienic living conditions, all exacerbated by increased risk of famine [52,53].

Overall, therefore, adiposity appears to represent a defining characteristic of hominin evolution in volatile environments. Enhanced plasticity in energy stores and metabolism contributed to the emergence of inflexible traits such as large brains, and life-history strategies such as sociality and delayed maturation. Fat represents a common energy currency that can fund diverse other physiological processes as required [5], and can therefore allocate energy to any of the four life-history functions, depending on what energy allocation strategy would optimize fitness at any given time. The fitness value of adiposity for contemporary humans in unpredictable environments is demonstrated by an ecological analysis, showing that in both sexes, triceps and subscapular skinfolds are larger in geographical locations with greater inter-annual temperature variability, a marker of ecological volatility [54].

## 3. The crafty genotype hypothesis

**Figure 2.**
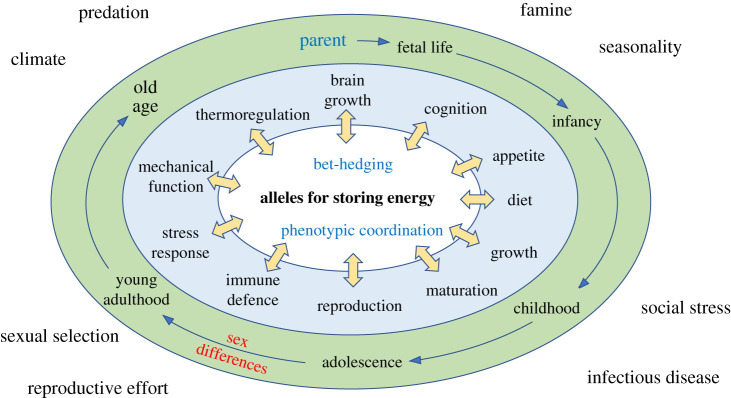
Schematic diagram illustrating how alleles associated with energy stores (adiposity alleles) relate to diverse physiological functions. The relative importance of these functions may vary through the life-course in both systematic ways (age, sex), and individual ways depending on the exposures experienced. (Online version in colour.)

In very rare cases, severe obesity can be attributed to chromosomal deletions or to mutations in specific alleles (monogenic obesity) inherited in Mendelian manner, whose effects are most evident through consanguineous mating [8]. While offering insight into metabolic pathways relevant to weight regulation, monogenic obesity has little relevance to the hypothesis presented here, which focuses on the polygenic basis of adiposity. The miniscule magnitude of effect of a typical adiposity allele may seem to indicate that such alleles are unable to respond to selection. The crafty genotype hypothesis presented here (figure 2) posits a more complex scenario.

First, it appears that adiposity alleles with large magnitudes of effect have been selected *against*. This may be an evolved correlate of enhanced plasticity: physiological systems that allow phenotype to respond sensitively to ecological conditions could be impaired if phenotype could be substantially changed by single alleles. A particular example of this is fetal adiposity, where elevated weight at delivery could cause maternal death from obstructed labour. This may explain why fetal fat deposition begins only in the third trimester [55], and why adiposity then increases substantially after birth [56–58].

Second, in stochastic environments, evolution inherently favours genetic bet-hedging—increasing phenotypic variability within a population, thereby increasing the likelihood that some individuals will always survive and reproduce, whatever the environmental state [59]. By distributing the genetic basis of variability in adiposity across numerous alleles, selection is unlikely to systematically eradicate any given allele from the gene pool over a short period, whatever the ecological shocks that occur. Consequently, the overall genetic variability in adiposity and its fitness-enhancing properties are resistant to selection, and are preserved in the population over time.

Third, the role of adiposity alleles in diverse physiological functions (figure 2) suggests that genotype-induced phenotypic variability is subject to coordination. Being fatter may only increase the Darwinian fitness of an organism if multiple other traits shift in a coordinated direction. For example, larger body size will require larger fat stores to survive a given period of negative energy balance, which in turn requires increased appetite [60]. Such associations may be bi-directional, and may also reflect either positive correlations across traits or negative correlations (trade-offs). For example, early reproduction in females may involve a trade-off between linear growth and fat accretion, as reported empirically [46,61] and supported through GWAS [62]. In this scenario, females trade-off early maturation against reduced adult body size (predicted to reduce offspring birth weight), but benefit from an increased capacity to fund lactation and infant growth from their relatively greater adult fat reserves [63].

Fourth, independent of adiposity alleles themselves, selection has also favoured sophisticated mechanisms of plasticity, through which energy stores respond sensitively through reaction norms, both to developmental exposures in early life, and to ongoing ecological stimuli and stresses through adult life [13]. The effect of adiposity alleles may thus interact in complex ways with life-course environmental exposures.

**Figure 3.**
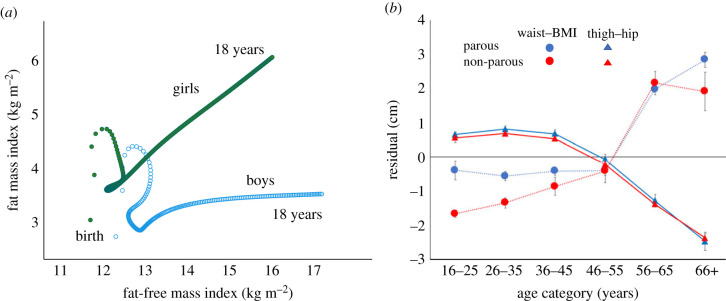
Age and sex variability in human body composition. (*a*) Fat mass index plotted against fat-free mass index from birth to 18 years, using the 50th centile of UK reference data [64,65]. Fatness is high in infancy, then declines before increasing again in mid-childhood, though with increasing sexual dimorphism. (*b*) Variability in fat distribution in Thai women. Residuals from regressing waist girth on BMI, and thigh girth on hip girth, are plotted against age group for parous and non-parous women. In both groups, waist-BMI increases and thigh–hip declines with age, indicating redistribution of body fat, though the data are cross-sectional. Data from the National Sizing Survey of Thailand. Redrawn with permission [78]. (Online version in colour.)

These properties of the crafty genotype relate to its resistance to external selective pressures and its coordination of diverse metabolic traits. This does not mean that adiposity does not evolve, rather the most fundamental factors that interact with these properties are age and sex. That the fitness value of fat varies with age in a sex-specific manner is evident both for total adiposity, adjusted for body size, and its anatomical distribution. Infants rapidly gain fat relative to height, before losing it in early childhood (figure 3*a*). Boys then show a peak in adiposity before the pubertal spurt in fat-free mass, while girls increase in more linear fashion to adulthood [64–66]. In every human population, young adults show major sexual dimorphism with higher adiposity and lower fat-free mass in females relative to males, reflecting both natural and sexual selective pressures [32,67]. However, the magnitude of these sex differences also varies with ecological conditions [67].

To understand why the distribution of fat as well as its amount varies with age, we must return to the ecological factor that is the strongest selective pressure, namely infectious disease. Mortality from infections is greatest in early life and older age [68], helping understand high levels of adiposity in infancy and the changing distribution of fat by age. The metabolic profile of fat varies markedly by adipose tissue depot. Broadly, abdominal or visceral adipose tissue is more metabolically active than peripheral depots, and a number of studies have linked visceral adiposity with immune function [69–72]. For example, visceral adipose tissue expresses complement genes more than peripheral adipose tissue [70]. However, the anatomical distribution of fat is not fixed, but rather varies in association with various factors.

As adults age, their fat distribution becomes more central and hence more immunogenic [73], a state termed ‘inflammaging’ [74]. In both sexes, abdominal fat accumulates with age while peripheral fat declines, independent of weight change [75–77]. Figure 3*b* illustrates profound changes in body shape associated with age in Thai women, stratified by whether they have reproduced or not [78]. Young parous women have low waist–BMI ratio and high thigh–hip ratio, indicating a peripheral fat distribution that is used during lactation [79]. Among women in their mid-40s, these traits reverse, indicating substantial redistribution of fat from peripheral to abdominal depots (though the data are cross-sectional). The same patterns are evident in non-parous women, indicating how the young adult body is prepared for reproduction even if it does not occur, and how fat is redistributed to ‘survival’ fat for immune function with older age, when adults can increase their fitness primarily through providing grandparental support [80–82].

That central fat generically funds immune function is indicated by an eco-geographical analysis, where across non-Western populations, a marker of greater pathogen burden was associated with lower subscapular but not triceps skinfold [83]. However, populations also show systematic geographic variability in their fat distribution [84]. Reflecting the importance of infectious disease as a selective pressure, the ‘variable disease selection hypothesis' assumes that the optimal distribution of body fat for immune defence may depend on which specific diseases pose the greatest risk, as individual pathogens create energetic stress in different components of physiology [48]. For example, intra-muscular lipid may be favoured to combat malaria, whereas visceral fat may help defend against gut-borne pathogens [48]. Whether such variability is genetic, or triggered by life-course exposures, is unknown.

The crafty genotype therefore enables fat to be moved around the body depending on age and sex [75–77], in order to maximize the fitness returns from storing energy. Moreover, maternal fat reserves can influence phenotype in the next generation. Energy stored in one generation (liquid capital) can buffer against ecological stresses confronting the offspring, and promote infant growth (somatic capital) [85]. For example, across 12 low- and middle-income countries, high maternal BMI showed protective effects against infant stunting [86], and another multi-country analysis showed similar associations, independent of dietary diversity and maternal wealth and education [87].

## 4. Varying the fitness value of fat in association with life-course experience

Beyond its overall ‘revaluing’ of the fitness value of fat by age and sex, the ‘crafty genotype’ also allows the value of fat to change according to life-course circumstances and experience, through a range of reaction norms. I focus here on three common exposures, namely low birth weight, child undernutrition, and low social status, while food insecurity is an additional example [88]. All of these initially cause ‘thrifty phenotypes’ [89] with reduced fat-free mass, with implications for survival and longevity. In turn, this may alter the valuation of fat stores. Although fat is an efficient means of storing energy, owing to its high energy density and low ‘access costs’ (energy required to accrete or oxidize fat) per gram, it is more costly to gain fat than fat-free tissue. Investing in fat is therefore a high-risk–high-reward strategy, especially for individuals that are already thin in terms of fat-free mass.

From the 1980s, infants born small were found to be at increased risk of cardiovascular disease, hypertension and type 2 diabetes, and also to have reduced overall survival [90–92]. Subsequent studies linked catch-up growth and adult adiposity as mediating mechanisms [93,94]. In adulthood, the ratio of fat to fat-free mass tends to be elevated following fetal undernutrition [95], but this can be attributed to persistent low fat-free mass, rather than high adiposity *per se*. The distribution of fat also tends to be more central, but again owing to reduced peripheral fat rather than extreme abdominal fat [96]. Such variability may be considered part of a broader adaptive strategy, whereby following reduced maternal investment in early life, the offspring prioritizes survival and early reproduction over growth and long-term metabolic health [46,96].

Similar patterns of development are evident among survivors of severe child undernutrition. In the short term, both wasting and stunting reduce linear growth, fat-free mass and fat mass, but over time, fatness tends to be preserved whereas deficits in height and fat-free mass persist [97,98]. Again, the distribution of fat appears more central, owing to depletion of peripheral fat [98].

Finally, low socio-economic status is associated with reduced fetal and infant growth [99] and low height and fat-free mass in childhood [100,101], though such associations may be less evident in adult life once fat-free mass is adjusted for the shorter height. In low-income settings, low socio-economic status may also be associated with low adiposity [102]; however, in middle- and high-income countries the relationship reverses, and those of low socio-economic status tend to show greater fat mass and a more central fat distribution [101]. This indicates that poverty drives exposure to both undernutrition in early life and the obesogenic niche at later ages [86]. In social animals in general, increased fat stores may have fitness value for subordinate individuals, compensating for their reduced opportunities to feed [103]. Studies of birds, for example, found that experimentally induced food insecurity increased fat deposition despite no apparent increase in energy intake, owing to compensatory reductions in other metabolic processes [104]. Such mechanisms are certainly plausible in humans, but the magnitude of their effect may only be reliably quantified if diet is controlled, a difficult undertaking in free-living humans.

In each of these cases, all representing forms of thrifty phenotype [89], exposure to nutritional constraint may result in a higher proportion of fat as weight and a more central fat distribution, detrimental to cardiometabolic health. This might be considered a simple life-history trade-off, whereby storing fat generates short-term benefits at a cost to longevity. However, some of these cardiometabolic costs may never be realized, as regardless of whether fat stores are high or low, exposure to undernutrition also shortens life expectancy [105,106]. If long-term costs are subject to ‘future-discounting’, then the short-term benefits of storing fat may be enhanced. Overall, therefore, these examples show how the fitness value of fat can change through shifts in both its metabolic benefits and its costs.

## 5. Conclusion

Evolutionary models of human obesity to date have been relatively simple, in assuming a constant association between adiposity alleles and selective pressures regardless of age or sex, and ignoring anatomical variability in fat deposition. The polygenetic basis of adiposity has been interpreted as it being non-conducive to selection except at extreme levels, as described in the dual intervention model [11].

The framework presented here offers a novel perspective on the polygenetic basis of adiposity. I argue that selection has favoured a crafty genotype, where the role of numerous alleles helps stabilize the genetic basis of phenotype in order to underpin enhanced plasticity, a strategy that is appropriate for a long-lived organism occupying volatile environments. The crafty genotype allows the fitness value of fat to vary in association with endogenous characteristics and life-course experience. The strategy of storing energy in fat has evolved to a highly sophisticated state, with contrasting fat depots and associated functions whose fitness values vary. This framework, highlighting the plasticity of adipose tissue biology, helps explain why weight gain is sensitive to numerous environmental factors, and why the health impact of obesity itself varies by age, sex, ethnicity and life-course experience. At the population level, the primary cause of trends in obesity is environmental, and has a profound economic basis [5,27].

## Data Availability

This article does not contain any additional data.
